# Exopolysaccharides Isolated from Hydrothermal Vent Bacteria Can Modulate the Complement System

**DOI:** 10.1371/journal.pone.0094965

**Published:** 2014-04-15

**Authors:** Anthony Courtois, Christian Berthou, Jean Guézennec, Claire Boisset, Anne Bordron

**Affiliations:** 1 Biotechnology and Marine Molecules Laboratory, IFREMER, Brest, France; 2 Cellular Therapy and Immunobiology of Cancer Laboratory, University Hospital of Brest, Brest, France; University of Leicester, United Kingdom

## Abstract

The complement system is involved in the defence against bacterial infection, or in the elimination of tumour cells. However, disturbances in this system contributes to the pathogenesis of various inflammatory diseases. The efficiency of therapeutic anti-tumour antibodies is enhanced when the complement system is stimulated. In contrast, cancer cells are able to inhibit the complement system and thus proliferate. Some marine molecules are currently being developed as new drugs for use in humans. Among them, known exopolyssacharides (EPSs) generally originate from fungi, but few studies have been performed on bacterial EPSs and even fewer on EPSs extracted from deep-sea hydrothermal vent microbes. For use in humans, these high molecular weight EPSs must be depolymerised. Furthermore, the over-sulphation of EPSs can modify their biological activity. The aim of this study was to investigate the immunodulation of the complement system by either native or over-sulphated low molecular weight EPSs isolated from vent bacteria in order to find pro or anti-activators of complement.

## Introduction

The complement system plays a very important role in the defence against infection, in antibody activity and in the clearance of antigen-antibody complexes from the bloodstream [1]–[3]. Its perpetual activation leads to inflammatory diseases creating irreversible tissue damages [4]. The complement system consists of approximately 35 different serum and cellular proteins, including positive and negative regulators that interact in a cascade. For example, many of the first components acting in the cascade are serine proteases that sequentially activate one another [5]–[7]. The complement system can be activated through three pathways: the classical, alternative and mannose-binding lectin (MBL) pathways. The classical pathway occurs mainly when a complex antigens and IgG (or IgM) antibodies binds to the first complement component C1q [8]. The alternative, or properdin, pathway is induced through contact with activated C3 that is deposited on a variety of surfaces, including pathogens such as viruses and fungi, but also on host cells in a variety of diseases such as auto-immune disorders or psoriasis [5], [9]. The MBL pathway initiates complement activation when notably lectins recognise mannose on the cell surface of pathogens such as bacteria [10]. Once activated, a cascade of events occurs until all three pathways converge at C3, ultimately leading to the assembly of a multi-protein complex (C5b6789) on a cell membrane. This is known as the membrane attack complex (MAC), and results in cell lysis. Among the key mechanisms and molecules involved in the innate immune response, glycosylation and glycans play essential roles in receptor recognition of ligands. Oligosaccharides interact with proteins of the complement system and play a major role in the stability, recognition and regulation of these proteins [11], [12].

Microorganisms can be considered as a renewable source and can be efficient producers of a large variety of bioactive molecules such as metabolites, proteins, peptides and novel exopolysaccharides (EPSs) [13]. Most bacterial EPSs have very complex and diverse structures that confer numerous functional properties. Over the past several years, there has been growing interest in the biological activity of microbial exopolysaccharides in regard to their anti-tumour activity [14]–[16], immunostimulatory activity [17]–[19], as well as their role in bone and tissue regeneration [20], [21] and anti-complementary activity [22].

In the present study, we evaluated the potential activity of two marine bacterial EPSs on the complement system. The first EPS, called GY785, is a high molecular weight (up to 10^6^ Da), branched, sulphated polysaccharide produced by *Alteromonas infernus*, a marine bacterium isolated from a deep-sea hydrothermal vent [23]. The second EPS, called HE800, is produced by *Vibrio diabolicus* another marine bacterium isolated from a deep-sea hydrothermal vent [24]; it has a linear backbone and a molecular mass of about 8⋅10^5^ Da. The latter EPS can be classified as a glycosaminoglycan due to some structural similarity with hyaluronic acid. This biopolymer has already shown very interesting biological properties in regard to bone and skin regeneration [20], [21]. The high molecular weight polymers were first depolymerised to decrease their viscosity, and to enhance their interaction with potential receptors or ligands. Furthermore, sulphation of the hydroxyl groups present on the polysaccharides can substantially modify their biological activity [25]. Thus, we assessed the effect of over-sulphated, low molecular weight (LMW) EPSs on the complement system. Interaction between the modified EPSs and the C1q protein of the complement system was studied and the results are reported and discussed.

## Material and Methods

### 2.1. Production, purification and characterization of native EPSs

The isolation, characteristics, production and purification of the GY785 and HE800 EPSs have been previously described [23], [24]. Briefly, exopolymer production was performed at 25°C in a 2-L fermenter (SGI-Inceltech, Toulouse, France) containing 1 liter of 2216E-glucose broth. Batch was inoculated at 10% (v/v) with a suspension of cells in exponential phase. The pH was adjusted and maintained at 7.2 by automatic addition of a 0.25 mol L^−1^ sodium hydroxide solution. The air flow was fixed at 30 L h^−1^ and the agitation rate from 200 to 1100 rev min^−1^. Bacterial growth was determined by measuring the culture turbidity at 520 nm. After 60 h of fermentation, bacterial cells were removed from the culture medium by centrifugation (15,000g, 80 min). The supernatant containing the excreted EPS was then purified by filtration through a cellulose membrane (0.7 µm) and then by ultrafiltration (100 kDa) before being freeze-dried and stored at room temperature away from light and moisture.

The monosaccharide residues of this both EPSs were analysed after acid methanolysis of the polymer and subsequent GC analyses as trimethylsilyl derivatives [26], [27]. The protein content was determined using Lowry method [28] with bovine serum albumin used as a standard. The sulphate content was determined by Fourier-transform infrared analysis [29]. Elemental analysis (C, H, N and S) was performed by the Central Microanalysis Department of the CNRS (Gif-sur-Yvette, France).

### 2.2. Depolymerisation of the EPSs

The EPSs were hydrolysed using radical depolymerisation, a procedure adapted from Nardella *et al*. [30]. Briefly, 400 mg of EPSs were dissolved in water (100 mL) and copper acetate was added (0.18 mM of Cu_2_OAc_4_). The temperature was maintained at 60 °C. A 0.14% (w/v) H_2_O_2_ aqueous solution was then added at a flow rate of 1 mL/min. The reaction pH was maintained at pH 7 by continuous addition of 10 N sodium hydroxide. The reaction was stopped after 2 h and the EPSs were reduced by NaBH_4_ (1/1, w/w). The excess of NaBH_4_ was eliminated by adding acetic acid. The precipitate formed was first filtered with a Büchner system using 3 µm microfiber filters. The residual copper was then eliminated on a Chelex 100 resin using water as an eluent (Bio-Rad, Hercules, CA, USA). The solution (called DR) was concentrated, desalinated by ultrafiltration with a 1 kDa cut-off membrane (Millipore, Molsheim, France) and then freeze-dried.

### 2.3. Sulphation of the LMW EPSs

The resulting depolymerised EPSs were chemically over-sulphated (called DROS) as previously described [31] with some modifications. Briefly, 1 g of dried LMW EPS was dissolved in 50 mL of double-distilled water and then applied to a cation exchange column (1×20 cm) of Amberlite IR 120H^+^ (Fluka Chemica, Saint-Quentin-Fallavier, France) equilibrated with double-distilled water. The column was then washed with water and the EPS fraction was collected, neutralised with a triethylamine solution to pH 5.5–6 and freeze-dried. The product was then dissolved in 100 mL of N,N,dimethylformamide (DMF) (Sigma-Aldrich) and mixed slowly for 2 h at room temperature then for another 2 h at 45 °C. An excess of pyridine-SO_3_ complex (5/1 (w/w) pyridine/EPS) was added and the reaction took place for 2 h at 45 °C. The reaction was stopped by the addition of an equal volume of ice-cold water. Finally, the solution was neutralised to pH 7.5 with a 2 M NaOH solution, concentrated, desalinated by ultrafiltration with a 1 kDa cut-off membrane (Millipore) and then freeze-dried.

### 2.4. Molecular weight determination of depolymerised EPSs

Each LMW EPS (2 mg/mL) was analysed by high-pressure size exclusion chromatography in 0.1 M ammonium acetate at a flow rate of 0.5 mL/min using a Superdex 200 column (Amersham, Saclay, France). Calibration was performed using pullulan standards [13], which are neutral glycans. Calculations of Mw (weight-average molecular mass), Mn (number-average molecular mass) and Ip (polydispersity index) were carried out using Aramis software (JMBS Développements, Le Fontanil, France).

### 2.5. Capacity of NHS (normal human serum) to lyse 50% of EA through the classical pathway (CH_50_ assay)

CH_50_, represents the NHS concentration that leads to lysis of 50% of antibody-sensitised sheep erythrocytes (EA). To determine the CH_50_ concentration, 800 µL of NHS, at different concentrations in VBS^2+^ [(4 mM veronal, 0.15 mM NaCl, 0.15 mM Ca^2+^, 0.5 mM Mg^2+^ (Sigma-Aldrich), pH 7.3] was incubated with 200 µL of EA at 10^8^ cells/mL for 45 min at 37 °C. EA were prepared by incubating sheep erythrocytes with rabbit anti-sheep erythrocyte antibodies (bioMérieux, Paris, France) as described by Kazatchkine [32]. Controls with 0% (L_0_) and 100% (L_100_) of lysis were obtained by incubating, in the same conditions, 800 µL of VBS^2+^ with 200 µL EA. After dilution in 2 mL of cold 0.15 M NaCl (except for L_100_, where 2 mL of double-distilled water (ddH_2_O) were added) and centrifugation, the residual released haemoglobin in the supernatant was determined by measuring the optical density (OD) at 414 nm [32].

### 2.6. Evaluation of the classical complement pathway activation

The ability of the LMW EPSs to activate the classical pathway of the complement system was measured using haemolytic assays in two steps (cf, above schema). First, various amounts of LMW EPSs (0 to 100 µg) were pre-incubated with 15 µL of NHS (at CH_50_ in VBS^2+^) for 45 min at 37 °C. If they can activate complement, consumption of complement molecules occurred. In the second step, a mixture of 100 µL of C1q, C4 or C2 deficient or depleted sera (Sigma-Aldrich, Paris, France) (1/40 (v/v) in VBS^2+^) and 100 µL of EA at 10^8^ cells/mL were added and incubated for another 45 min at 37 °C. If no lysis of EA was observed, this indicated that activation of complement was achieved previously; the percentage of EA lysis inhibition corresponding to the percentage of activation. The controls L_0_ and L_100_ were obtained as described above. After dilution with 2 mL of cold 0.15 M NaCl and centrifugation, the amount of haemoglobin released was assessed by measuring the OD (Optical Density) of supernatants at 414 nm. A positive control of the reaction was obtained by incubating aggregated IgG (0–100 µg) in the same experimental conditions [33]. Results were presented as percentage of activation corresponding to percentage of EA lysis inhibition, then the percentage of consumption of complement molecules by EPSs in CH_50_ NHS.

### 2.7. Evaluation of the inhibited-complementary activity of depolymerized EPSs

**Figure 1:**
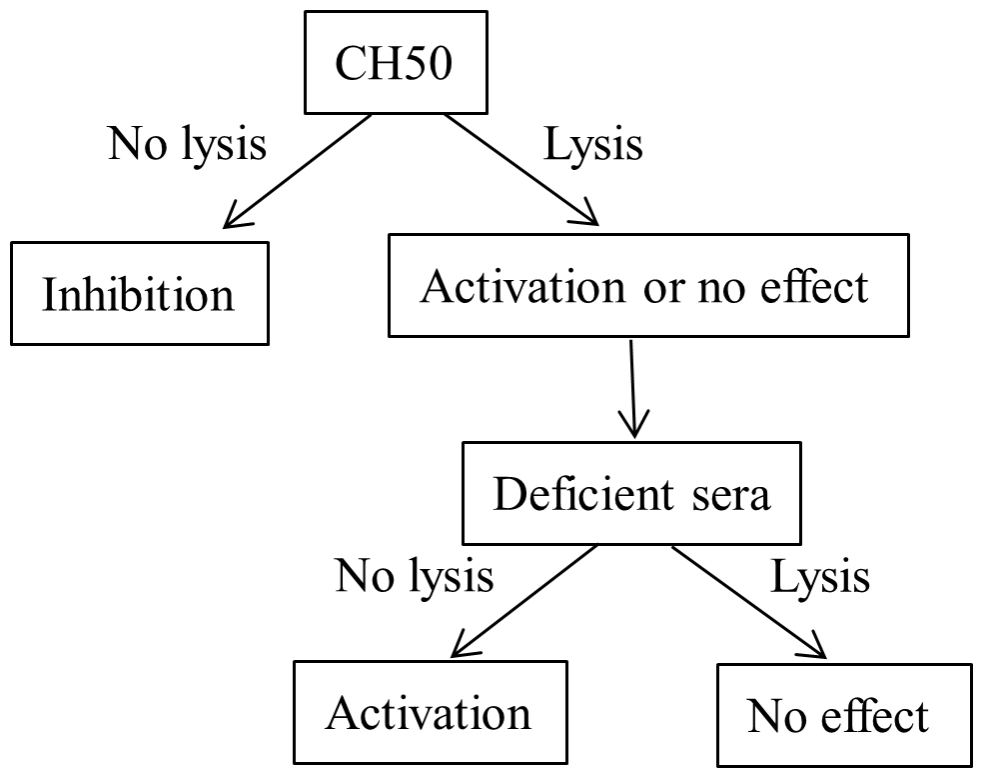
Evaluation of the classical complement pathway activation or inhibition.

The inhibited complement activation of the depolymerised and over-sulphated LMW EPS was assessed using haemolytic assays. The ability of NHS to lyse EA through the classical pathway was assessed with various amounts of LMW EPSs. In this process, 350 µL of NHS (1/100 in VBS^2+^) were incubated with 450 µL of VBS^2+^ buffer containing 0–10 µg of LMW EPS and 200 µL of EA at 10^8^ cells/mL, for 45 min at 37°C. After addition of 2 mL of 0.15 M NaCl and centrifugation, the residual CH_50_ units of the supernatant were determined by the measuring the OD at 414 nm. Positive control of the reaction was obtained by incubating 0–10 µg of a LMW sulphated fucoidan (called FTDR) in the same experimental conditions. The Figure 1 resumes the evaluation of the classical complement pathway activation or inhibition.

### 2.8. Surface plasmon resonance (SPR) analysis

C1q was immobilized on a CMD 200 l sensor chip (Xantec, Duesseldorf, Germany) in PBS buffer (running buffer) via its primary amino groups using a standard EDC/NHS-mediated amine coupling procedure. In this procedure, 100 µL of various dilutions of GY 785 DROS in PBS buffer (running buffer) were injected at a flow rate of 100 µL/min. The sensor surface was regenerated by injection of 10 mM NaOH in 2 M NaCl. The response was monitored over time (sensorgram) at 25°C. All sensorgrams were processed using a double referencing method [34]. First, the responses from the control cell were subtracted from the binding responses collected over the reaction cell to correct for bulk refractive index changes. Second, the response from the blank injection was subtracted to remove responses observed due to the change in running buffer and other system artefacts. The sensorgrams obtained were fitted using a 1∶1 interaction model with drifting baseline, using the Biacore evaluation sofware (version 3.2).

### 2.9. Statistical analysis

All results are expressed as means ± standard error. Statistical differences between experimental groups were determined by analysis of variance (ANOVA) and individual means were compared with a Student's *t*-test using InStat (GraphPad Software, San Diego, CA, USA). Values of *p*<0.05 were considered statistically significant.

## Results and Discussion

### 3.1. Production of LMW EPSs

Classical depolymerisation methods include chemical hydrolysis, ozonolysis, oxidation or specific enzymatic depolymerisation. An alternative method, radical depolymerisation, generally preserves the structure of the repetition unit of an EPS. In radical depolymerisation, free radicals are formed via the hydrogen peroxide-cupric redox system, leading to good yield under mild experimental conditions [13], [26]. The molecular weights of the depolymerised EPSs are listed in Table 1. Results are expressed in equivalent pullulans, used as the calibration standard. Gas chromatography (GC) analysis of monosaccharides as *per-O*-trimethylsilyl methylglycosides and the determination of monosaccharide molar ratios were carried out, demonstrating that the monosaccharide composition was similar before and after depolymerisation. For GY785, the monosaccharide ratio was found to be 4∶2∶1∶2 glucose:galactose:galacturonic acid:glucuronic acid and for HE800, the composition was 1∶1∶2 N-acetyl glucosamine:N-acetyl galactosamine:glucuronic acid. Depolymerisation did not alter the osidic composition of either EPS. The LMW EPS fractions had the same sulphate-to-total sugar ratios as the high molecular weight EPSs. The percentage of proteins present with the EPS was determined using the Lowry colorimetric assay. Depolymerisation eliminated most of the proteins.

**Table 1:** Molecular weights (Mw), polydispersity index (Ip), yield of sulphate groups and percentage of proteins in native, depolymerized (DR) and over-sulphated (DROS) GY785 and HE800 EPSs, using pullulans as standards.

	Mw (Da)	Mn (Da)	Ip (Mw/Mn)	Sulphates^(1)^ % w/w	Sulphates^(2)^ % w/w	Proteins (%)
Native GY785	1.4⋅10^6^	1.2⋅10^6^	1.2	10	9	13
GY785 DR	1.7⋅10^4^	6.0⋅10^3^	2.8	10	8	<1
GY785 DROS	2.3⋅10^4^	8.8⋅10^3^	2.7	42	37	<1
Native HE800	7.4⋅10^5^	6.7⋅10^5^	1.1	0	0	1
HE800 DR	2.9⋅10^3^	1.3⋅10^4^	2.2	0	0	<1
HE800 DROS	2.7⋅10^4^	1.9⋅10^4^	1.4	34	29	<1

Mw, weight-average molecular mass; Mn, number-average molecular mass; sulphates^(1)^: percentage of sulphate groups determined using Fourier-transform infrared analysis; sulphates^(2)^: percentage of sulphate groups determined using elemental analysis.

### 3.2. Sulphation of LMW EPSs GY785 and HE800

The over-sulphated molecules, designated HE800 DROS and GY785 DROS, were analysed for their chemical composition, and the sulphate content was estimated using Fourier-transform infrared analysis and elemental analysis and compared to the native EPS and LMW forms. Both methods gave the same, results, as shown in Table 1. GY785 DR and the GY785 DROS showed sulphate contents of 10% and 42%, respectively, whereas the sulphate content of HE800 DROS was 34% (w/w). Gas chromatography analysis of monosaccharides as *per-O*-trimethylsilyl methylglycosides, carried out after over-sulphation, showed that over-sulphation did not modify the osidic composition of either EPS.

### 3.3. Complement activation properties of the LMW EPSs

As glycosylation can influence complement activity, the classical pathway of complement system was activated via contact with NHS, used as the complement source, with EA, leading to the lysis of the EA.

**Figure 2:**
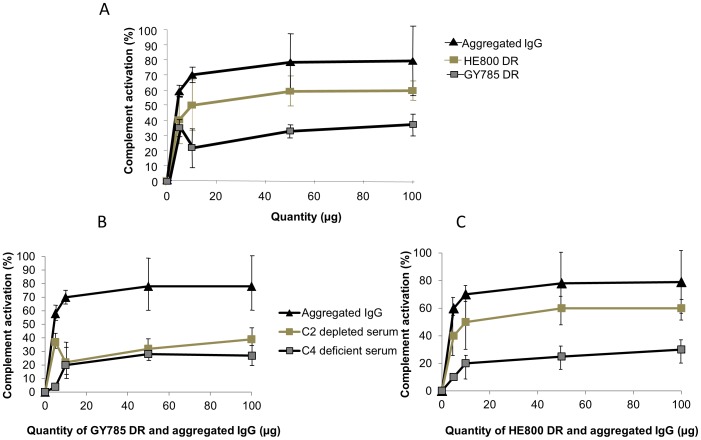
Activation of the classical complement pathway by depolymerised GY785 and HE800. Activation was measured by the capacity of normal human serum (NHS) incubated with various amounts of GY785 DR or HE800 DR, to restore the haemolytic activity of serum deficient in complement proteins C1q (**A**), C4 (**B**) and C2 (**C**). Aggregated IgG were used to validate the experimental model using C2-deficient serum. Each point represents the mean (SEM) from 3 to 6 determinations.

To assess the ability of the GY785 DR and HE800 DR to activate the complement system in NHS, we assessed how occurred complement activity of serum deficient or depleted in proteins C1q, C2 and C4 of the complement system. First, the CH_50_, representing the NHS dilution that leads to lysis of 50% of the EA, was determined. In our experiments, the CH_50_ was reached for a 1∶100 dilution of NHS. Then, increasing amounts of LMW EPS (0-100 µg) in NHS solution at the CH_50_ concentration were incubated with deficient or depleted serum and complement activation was determined. In all experiments (Figure 2), aggregated IgG were used as a positive control, demonstrating an ability to activate the complement system, reaching 70% activation for 100 µg of proteins.

Our results demonstrate that HE800 DR and GY785 DR activated the classical complement system (Figure 2A), HE800 DR in more important way. These results were confirmed in Figures 2B and 2C. In fact, for GY785 DR the plateau was reached at 38% activation for a dose of 100 µg (Figure 2B). As shown in Figure 2C, the amount of activation of the complement system in the presence of HE800 DR in veronal buffer (VBS^2+^) increased rapidly with the amount of polysaccharide and reached a plateau that corresponded to 60% activation for 50 µg of HE800 DR. We can note that results obtained for GY785 DR in three different sera were homogenous this was not the case for HE800 DR. In fact, for C4 deficient serum, complement activation was lower. Different explanations could be suggested such as conformation of EPSs and its fixation on complement molecule but must to be explored more precisely. As mentioned above, various glycans have been described to activate the classical pathway of the complement system [35]. For example, hyperacute rejection in pig-to-primate xenotransplantation is due to a carbohydrate antigen, called the Gal-α1,3)Gal oligosaccharide, and humans are predisposed to produce large amounts of a natural antibody called ‘anti-Gal’, which binds specifically to α-Gal epitopes on glycolipids and glycoproteins [36], [37] followed by activation of the complement system [38]. Based on these observations, we have previously shown that when the Gal-α1,3)Gal oligosaccharide is coupled to a therapeutic antibody, there is an increase in its efficacy [38]. ABO-incompatible blood transfusion shares several similarities with hyperacute rejection in xenotransplantation [39]. Lipopolysaccharides can also activate the complement system by directly recruiting the C1q protein [40]. This study is the first report of a correlation between a marine bacterial exopolysaccharide and direct complement activation, involving in particular HE 800 DR, whose structure has some similarities with hyaluronic acid. More studies must be carried out to determine precisely how this polysaccharide activate the complement system, particularly in its interaction with the C1q protein.

### 3.4. Complement inhibition properties of sulphated LMW EPSs

**Figure 3:**
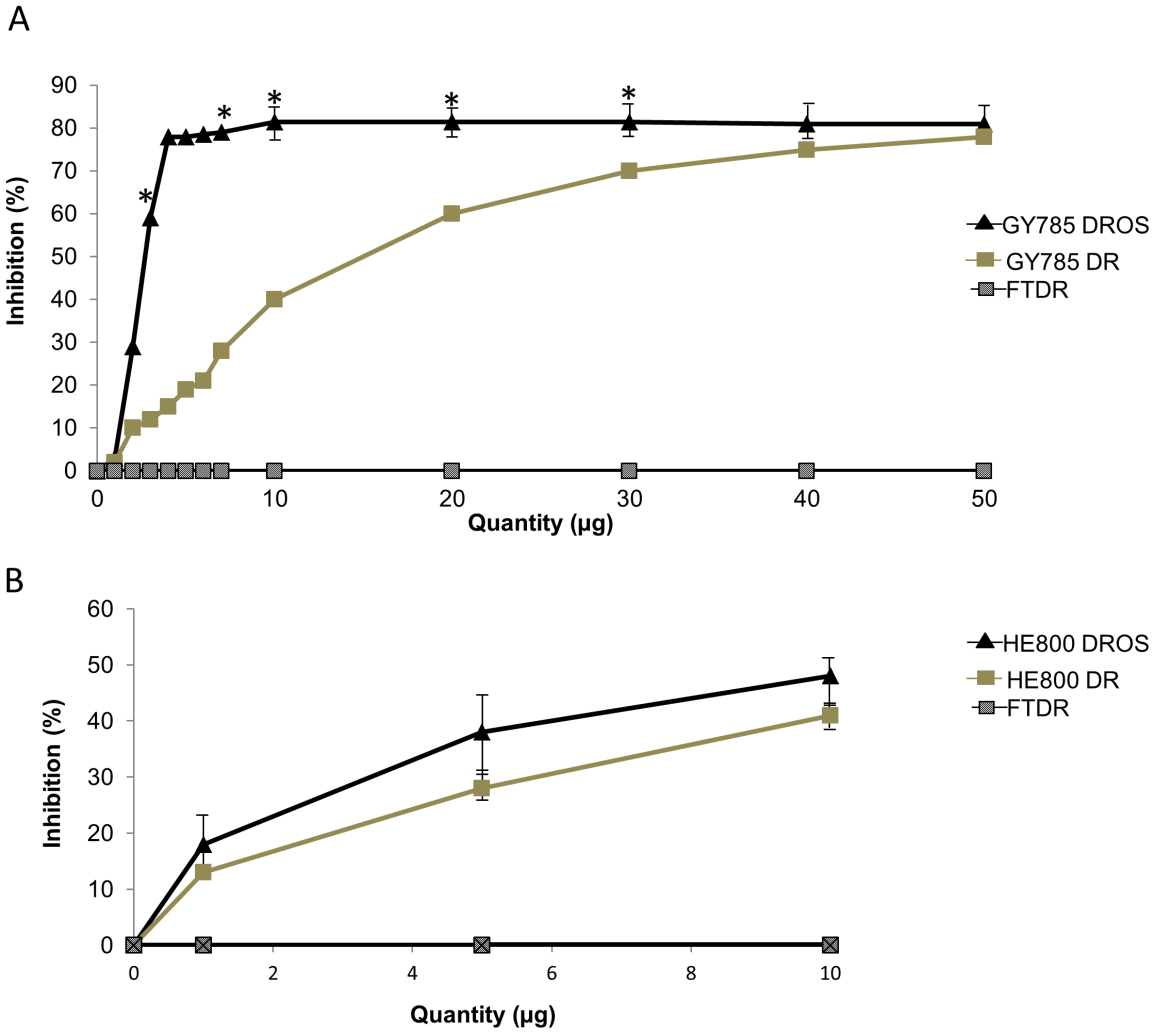
Inhibition of complement activation in normal human serum by depolymerised GY785 DR, over-sulphated GY785 DROS (A), depolymerised HE800 DR, over-sulphated HE800 DROS (B) and FTDR. Inhibition of the classical pathway was evaluated by measuring antibody-sensitised sheep erythrocyte haemolysis. The amount of each polysaccharide is indicated for a 100-fold diluted NHS prior to addition of erythrocytes. Each point represents the mean (shown with standard error bars) from four determinations, statistically analysed using Student's *t*-test. * indicates significant differences (p<0.05) between GY785 DROS and FTDR.

Sulphated glycosaminoglycans [41] are well known to inhibit the complement system *in vitro*. We investigated the role of the sulphate groups on the EPS glycan structure in regard to their capacity to inhibit the complement system, particularly via the classical pathway. Over-sulphated LMW EPS (GY785 DROS and HE800 DROS) showed inhibited complement activation as indicated by a decrease in EA lysis (Figures 3A and 3B). The inhibition activity was compared to that of an LMW fucoidan (FTDR) extracted from the brown alga *Ascophyllum nodosum* [42] and used as a positive control in the experiment. This LMW polysaccharide is naturally sulphated (35% (w/w) sulphate content) with a molecular weight of 1.8⋅10^4^ Da. It has already been described to have complement inhibiting activity in the same experimental conditions [42], [43].

More specifically, we found that GY785 DROS inhibited, in a dose-dependent manner, the classical pathway of the complement system (Figure 3A). This capacity increased very rapidly with small amounts of polymer and reached a plateau corresponding to 78% inhibition for a quantity of 10 µg of GY785 DROS compared to 41% with the same quantity of FTDR. The capacity to inhibit 50% of the complement system was obtained using 750 ng of GY785 DROS. GY785 DROS thus inhibits the complement system more efficiently than FTDR does, particularly at low concentrations. Furthermore, we found that HE800 DROS also has complement inhibiting effect (Figure 3B), whereas the non-sulphated LMW EPS had no effect on inhibition. HE800 DROS inhibition of the complement system was similar to FTDR inhibition activity, with an inhibition of 48% and 41% obtained with 10 µg of HE800 DROS and FTDR, respectively. In addition, the capacity to inhibit 50% of the complement system was obtained using 2.5 and 3 µg of HE800 DROS and FTDR, respectively.

These results suggest that the capacity to inhibit the complement system depended mainly on the sulphate groups, but also on polysaccharide structure (conformation, monosaccharide composition and physico-chemical properties).

### 3.5. Interaction of depolymerised and sulphated LMW GY785 and HE800 with the C1q complement protein

**Figure 4:**
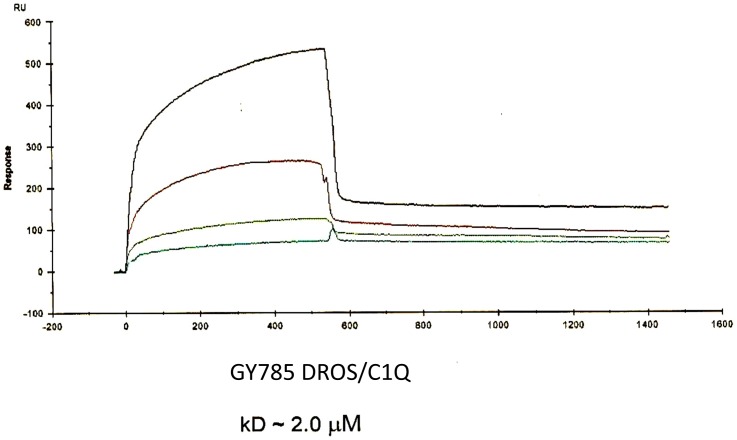
SPR sensorgrams of C1q-GY785 DROS interactions. Concentrations of GY785 DROS (from top to bottom) were 100, 50, 25, and 6.5 µM, respectively.

As described above, activity of fucoidan is due to complement inhibition via interaction with the C1q protein [42], [44]. Other studies have been performed on sulphated polysaccharides showing interaction with C1q protein and resulting in inhibition of complement system [45]. To demonstrate an interaction between the over-sulphated polysaccharides and serum proteins, it was not possible to characterise an interaction specifically with C1q. To better understand the physical association of C1q with GY785 DROS, we observed their interaction using surface plasmon resonance (SPR). SPR measures biomolecular interactions in real-time in a label-free environment, providing both equilibrium and kinetic information on the formation of complexes. SPR has been used to study the interactions between various complement proteins [46]. However, SPR studies on complement-protein interactions with sulphated polysaccharides are very rare [47], [48]. As complement inhibition was highest observed with GY785 DROS, only the interaction between this sulphated EPS with C1q was analysed by SPR. C1q was immobilised via its unsubstituted amino groups. Dextran sulphate, used as a negative control, did not interact with C1q (data not shown), but a specific response was monitored with GY785 DROS (Figure 4). The simultaneous fit of the association and dissociation phases, using a 1∶1 Langmuir model, allowed us to the determine a binding affinity value (KD) of about 2 µM, indicating the formation of a stable complex between GY785 DROS and the C1q complement protein.

A glycosaminoglycan chondroitin 4-sulfate proteoglycan secreted by human B lymphocytes has been proposed to act as a physiological inhibitor of C1q through the inhibition of C1 assembly [45]. More recently, fucoidan (as for FTDR) has been described to protect C1q from trypsin cleavage, indicating that fucoidan binds C1q at two different sites, one in the globular domain and the other in the stem, in the vicinity of the interaction site with C1s-C1r-C1r-C1s. This interaction is also responsible for the inhibition activity of fucoidan [44]. Based on these results, we propose that the sulphate groups on polysaccharides confer the ability to inhibit C1 by promoting an interaction with the C1q protein via the same domain as that observed in fucoidan. This protein-polysaccharide association probably involves ionic interactions between positive residues of this domain and the sulphate groups of the polysaccharide.

## Conclusions

Biological products on complement activity were obtained from new marine bacterial EPS originating from an extreme environment, following free radical depolymerisation and sulphation. A novel activator of the complement system was discovered. We demonstrated that sulphation of these polysaccharides confers inhibition of complement activity, mainly for GY 785 DROS. These data open very interesting perspectives for the treatment of diseases caused by deregulation of the immune system and over-activation of the complement system.
